# The Impact of the Histologic Types of Lung Cancer on CBC-Derived Inflammatory Markers—Current Knowledge and Future Perspectives

**DOI:** 10.3390/jcm14093038

**Published:** 2025-04-28

**Authors:** Claudia Raluca Mariean, Oana Mirela Tiucă, Alexandru Mariean, Tiberiu-Bogdan Szekely, Raluca Niculescu, Adrian Horatiu Sabau, Cristina Flavia Al-Akel, Ovidiu Simion Cotoi

**Affiliations:** 1 Doctoral School of Medicine and Pharmacy, George Emil Palade University of Medicine, Pharmacy, Science, and Technology of Targu Mures, 540142 Targu Mures, Romania; 2 Pathophysiology Department, George Emil Palade University of Medicine, Pharmacy, Science, and Technology of Targu Mures, 540142 Targu Mures, Romania; 3 Department of Radiology, Targu Mureș County Emergency Hospital, 540142 Targu Mures, Romania; 4 Dermatology Department, George Emil Palade University of Medicine, Pharmacy, Science, and Technology of Targu Mures, 540142 Targu Mures, Romania; 5 Dermatology Clinic, Mures Clinical County Hospital, 540342 Targu Mures, Romania; 6 Pulmonology Clinic, Mures Clinical County Hospital, 540103 Targu Mures, Romania; 7 Department of Oncology, George Emil Palade University of Medicine, Pharmacy, Science, and Technology of Targu Mures, 540142 Targu Mures, Romania; 8 Department of Oncology, Clinical County Hospital Mures, 540141 Targu Mures, Romania; 9 Pathology Department, Mures Clinical County Hospital, 540011 Targu Mures, Romania; 10 Department of Pediatric Cardiology, Emergency Institute for Cardiovascular Diseases and Transplantation of Târgu Mureș, 540136 Targu Mures, Romania

**Keywords:** lung cancer, histology, inflammation, CBC-derived inflammatory parameters, smoking, COPD

## Abstract

**Background/Objectives**: The analysis of the complete blood count (CBC)-derived inflammatory indexes across different histological subtypes of lung cancer supports the early detection of tumor-induced inflammation and has a good predictive value for severity in cancer patients. The main objective of this article was to assess the variations in CBC-derived inflammatory markers across different histologic subtypes of lung cancer, with the final goal of identifying specific predictors of severity for each histologic subtype of lung cancer. **Methods**: We conducted a retrospective descriptive study that included 202 patients diagnosed with lung carcinoma at the Clinical County Hospital Mureș. The analyzed parameters were as follows: the histological type, the stage of the tumor, patients’ general data, and associated comorbidities. In addition, nine CBC-derived inflammatory indexes, like the neutrophil-to-lymphocyte ratio (NLR), derived neutrophil-to-lymphocyte ratio (d-NLR), monocyte-to-lymphocyte ratio (MLR), platelet-to-lymphocyte ratio (PLR), eosinophil-to-neutrophil ratio (ENR), eosinophil-to-monocyte ratio (EMR), systemic inflammatory index (SII), systemic inflammatory response index (SIRI), and aggregate index of systemic inflammation (AISI), were analyzed as predictors of severity and correlated with histologic findings. **Results**: The predictors of severity differed across the histologic subtypes. SIRI, d-NLR, and age were predictors of severity in adenocarcinoma patients, while the d-NLR, ENR, leukocyte, and neutrophil count predicted severity in squamous cell carcinoma. For SCLC patients, AISI, SIRI, SII, d-NLR, EMR, ENR, MLR, leukocyte count, lymphocyte count, neutrophil count, platelets count, COPD, smoking, and male gender were predictors for severity. **Conclusions:** Understanding the complexity and variations in the inflammatory response across different histologic types of lung cancer can personalize treatment regimens and target specific abnormal cellular lines, thus improving the outcome of this highly deadly condition.

## 1. Introduction

Lung cancer, a global healthcare concern, is the second most commonly diagnosed type of malignancy and the leading cause of death in both males and females worldwide, with a 5-year survival rate of less than 15% [1]. In 2020, there were almost 2.2 million newly diagnosed cases of lung cancer and 1.8 million deaths related to it, making lung cancer responsible for up to 13.8% of all cancer deaths [2,3]. Considering the above-mentioned aspects, there is an increased need to identify efficient modalities for the early diagnosis and proper management of lung cancer patients.

In 2010, the National Lung Screening Trial (NLST) mentioned a 20% reduction in the mortality associated with lung cancer for high-risk patients screened with low-dose computed tomography (CT) scans. However, the same trial stated that the rate of over-diagnosis using CT scans was up to 18%, raising awareness about the associated radiation-exposure risks and increased anxiety in patients unnecessarily screened multiple times [4]. In addition to CT scans, a new technique that does not use ionizing radiation has emerged in recent years as an alternative to CT scans, particularly for pregnant women and the pediatric population [5]. Studies showed that MRI can be used as a potentially effective screening tool for lung cancer patients, which is similar to a low-dose CT scan (LDCT) but with decreased false-positive rates and no radiation exposure. In addition, magnetic resonance imaging (MRI) might be used to identify imaging-derived biomarkers that could stratify lung cancer patients according to the associated risk. In particular, diffusion-weighted imaging (DWI) reflecting tumor cellularity allows for the calculation of the apparent diffusion coefficient (ADC), a quantitative measure of tissue diffusivity that has been used to differentiate lung cancer subtypes. Although multiple challenges must be addressed before integrating MRI into routine clinical practice, it has been proposed as a useful and important tool for the early diagnosis of lung cancer patients, without the associated risks of ionizing radiation exposure encountered in CT scans [6].

Histology has been proposed as a potential prognostic factor in lung cancer patients, as different histologic types of lung cancer have distinct characteristics. These characteristics influence chemotherapeutic drugs’ transport, metabolization, bioactivation, and the predicted response to different treatment regimens [7,8]. Still, until now, there has been no agreement regarding the precise impact of histology on cancer-associated inflammation.

Histologically, lung cancer can be classified into small-cell lung carcinoma (SCLC), diagnosed in up to 10–15% of cases, and non-small cell lung carcinoma (NSCLC), the most frequent histologic type, diagnosed in up to 80–85% of cases. NSCLC can be further classified into three main histologic subtypes with different characteristics: lung squamous cell carcinoma (SCC), large cell carcinoma (LCC), and adenocarcinoma (AdC) [3]. Squamous cell carcinoma originates from the bronchial epithelium of the central larger airways. It follows a multistep development pathway, from preinvasive squamous metaplasia to squamous dysplasia and, in the end, carcinoma in situ (CIS) [9,10]. The pathophysiology of these neoplastic alterations involves numerous molecular changes, like the deregulation of cell proliferation (cyclin E and D1), apoptosis (Bcl-2), and the loss of heterozygosity at the 3p21 and p 53 mutation [11,12]. Adenocarcinomas, the most frequent NSCLC subtype, are more peripheric tumors originating from the alveolar or bronchiolar epithelium [11]. Adenocarcinomas also follow a multistep development, from atypical adenomatous hyperplasia (AAH) to adenocarcinoma in situ (AIS) [10]. The molecular alterations of adenocarcinomas are not as well defined as those encountered in squamous cell carcinoma. For smokers, alterations in v-Ki-ras2 Kirsten Rat Sarcoma viral oncogene (*KRAS*) signaling pathways are cited, while for non-smokers, alterations in epidermal growth factor receptor (*EGFR*) pathways are encountered [11]. Large cell carcinoma (LCC) is the least commonly encountered among the leading three histologic types of NSCLC and has no well-defined criteria for diagnosis. LCC accounts for 3% of all lung cancer cases and is frequently large, partially with associated necrosis, and composed of nests and sheets of large cells with prominent nucleoli. In addition to the main three NSCLC histologic subtypes, adenosquamous carcinoma is also mentioned. It can be diagnosed with certainty only in surgically resected fragments of the tumor and represents less than 5% of all lung cancers [13,14]. It comprises at least 10% of glandular and squamous differentiation [8]. Regarding SCLC, studies enunciated that it usually surpasses the multistep pathway and originates directly from an epithelial structure with minimal atypia, with genetic alterations being cited as one of the main pathophysiological explanations [11,15].

Diagnosed most often in late advanced stages (stages III and IV), lung cancer patients have a poor prognosis and a decreased overall survival rate. In recent years, a link between the process of carcinogenesis, tumor proliferation, dissemination, and associated local and systemic inflammation has been highlighted [16]. Persistent chronic inflammation leads to regional changes in the tumor immune microenvironment (TIME) and to systemic effects like the increased production of cytokines (interleukin-6 (IL-6)), interleukin-1 (IL-1), the macrophage colony-stimulating factor, an impaired cellular division rate, DNA damage, an increased cell apoptosis and angiogenesis [16,17,18]. Moreover, up to 15% of cancer-related mortality is directly linked to unresolved, persistent chronic inflammation [19].

In light of these aspects, as there is an increased need for a better understanding of the inflammatory response associated with NSCLC and SCLC, biomarkers might represent an important direction. Biomarkers provide an essential overview of the patient’s tumor characteristics, general and nutritional status, and potential response to therapeutic interventions [20]. In addition, identifying cost-efficient lung cancer biomarkers can improve patients’ early detection and risk classification.

Hematological parameters are non-invasive and accessible tools that can assess the patient’s immune status, identify high-risk mortality patients, and evaluate cancer prognosis [21]. The complete blood count (CBC), a routinely inexpensive investigation, can detect hematological parameters that assess the degree of associated inflammation and have a good predictive value for the outcome of cancer patients. Considering the complexity of immune reactions and cancer-associated inflammatory changes, a single indicator might not be enough to properly evaluate the associated inflammatory response in cancer patients [22].

In recent years, a number of studies have addressed the importance of inflammatory biomarkers for lung cancer patients’ prognosis. A study published by Winther-Larsen et al. in 2022 highlighted that the use of the Aarhus composite biomarker score (ACBS), which includes albumin, C-reactive protein, neutrophil counts, lymphocyte counts, and hemoglobin, was the optimal score to be used in NSCLC patients, while in SCLC patients, the modified Glasgow Prognostic Score (mGPS) and Combined NLR and Glasgow Prognostic Score (CNG) were significant [23]. A study published by Rice et al. in 2021 cited that NSCLC patients had significantly higher levels of systemic inflammation markers, such as NLR, the platelet-to-lymphocyte ratio (PLR), and the systemic inflammation index (SII), compared to SCLC patients. Elevated levels of these markers were associated with a worse progression-free survival (PFS) and overall survival (OS) in NSCLC but not in SCLC [24]. In addition, methylation-derived NLR (mdNLR) has been associated with a higher mortality in SCLC but not in NSCLC [25].

In addition, as stated before, histology seems to play a crucial role in the appropriate management of lung cancer patients, as different histologic types have unique characteristics that may highly impact the clinical outcome of lung cancer patients. Therefore, this study aimed to assess the relationship between the histological subtypes of both NSCLC and SCLC with nine CBC-derived inflammatory parameters at the time of the initial diagnosis and their use as predictors of severity for each histologic subtype. We looked into common and distinct aspects of the inflammatory response encountered in different histologic subtypes of lung cancer to identify the best predictors of severity for each histologic type of SCLC and NSCLC. Although the importance of inflammatory biomarkers was addressed, as stated before, by previous researchers, the main strength of the current study is based on the important relationship between the variations in different CBC-derived inflammatory biomarkers and their use as severity predictors and the histological subtypes of lung cancer encountered in our study population.

Nowadays, in the era of personalized medicine, identifying and using accessible predictive biomarkers reflecting histological changes can highly personalize the patients’ clinical approach and influence the therapeutic strategies, risk classification, and overall prognosis of this highly deadly condition.

## 2. Materials and Methods

### 2.1. Data Sources and Patients Included in the Study

We conducted a retrospective descriptive study, including 202 patients diagnosed with lung carcinoma between 1 January 2019 and 31 December 2023 at the Clinical County Hospital Mures, Târgu Mureș, Romania.

The inclusion criteria were as follows: (1) a histopathological diagnosis of lung carcinoma, (2) available selected laboratory data for analysis, (3) patients aged > 18 years old, (4) patients without associated active infections at the time of diagnosis, and (5) patients without other associated malignancies at the time of diagnosis.

The exclusion criteria included the following: (1) patients without a histopathological confirmation of the diagnosis of lung carcinoma, (2) patients without the selected laboratory data needed for analysis, (3) patients aged < 18 years old, (4) patients with associated active infections at the time of diagnosis, and (5) patients with other associated malignant disorders at the time of diagnosis.

This study was performed according to the Declaration of Helsinki, which was approved by the Ethics Committee of the Clinical County Hospital Mureș (approval 20419/15 December 2023).

### 2.2. Definition of Analyzed Parameters

The analyzed parameters included the following:

- The histological type of lung carcinoma: NSCLC (adenocarcinoma, squamous cell carcinoma, adenosquamous carcinoma, and NSCLC not otherwise specified (NOS)) and SCLC.
  Biopsies are needed to identify the histological type of the tumor. In our study group, tissue biopsies were performed, as described below. The primary method used was the transbronchial biopsy (TBLB), using a flexible bronchoscope via the transoral route for central tumors. For tumors that could not be accessed with the aid of bronchoscopy, a percutaneous transthoracic lung biopsy (PTLB), in which a needle was inserted through the patient’s chest wall with the assistance of CT guidance in the suspected area, was also performed for our study population.
- The stage of the tumor at diagnosis: The patients’ tumoral stage at diagnosis was established taking into consideration the TNM classification of malignant tumors, where T describes the primary tumor site and size, N describes the involvement of the regional lymph nodes, and M describes the presence of distant metastasis. The 8th edition of the TNM grading system was used for the proper tumor staging of the study population, as it is the latest version published in the scientific literature and provides the best characterization of the tumor staging [26].
- Parameters derived from the complete blood count (CBC) of the patients (neutrophils count, lymphocytes count, leucocytes count, monocytes count, platelets count, and eosinophils count)
- The following CBC-derived inflammatory indexes: Neutrophil-to-lymphocyte ratio (NLR); derived neutrophil-to-lymphocyte ratio (d-NLR); monocyte-to-lymphocyte ratio (MLR); platelet-to-lymphocyte ratio (PLR); eosinophil-to-neutrophil ratio (ENR); eosinophil-to-monocyte ratio (EMR); systemic inflammatory index (SII); systemic inflammatory response index (SIRI); and aggregate index of systemic inflammation (AISI). The detailed formulas of the included CBC-derived inflammatory indexes are displayed in Table 1.
- Data regarding the living environment (urban/rural), the gender, the age of the patients at diagnosis, exposure to tobacco smoke, and the presence of COPD as a comorbidity for the current disease
- BMI was calculated using the following formula: BMI = kg/m^2^. Based on BMI, patients were classified as underweight (BMI < 18.5 kg/m^2^), normal weight (BMI between 18.5 and 24.99 kg/m^2^), overweight (BMI between 25 and 29.99 kg/m^2^), grade I obesity (BMI between 30 and 34.99 kg/m^2^), grade II obesity (BMI between 35 and 39.99 kg/m^2^), and grade III obesity (BMI > 40 kg/m^2^).

### 2.3. Statistical Analysis of Data

The statistical analysis was performed using the MedCalc software, version 23.0.2. Data are expressed as mean ± standard deviation for parametric data and as median with a 95% confidence interval for non-parametric data. Normality was tested using the Shapiro–Wilk test. Logarithmic ANOVA was used to compare groups, with a subsequent post hoc Dunn–Bonferroni test, when applicable.

Spearman’s rank correlation was used for non-parametric data, and Pearson’s correlation was used for normally distributed data when appropriate.

Logistic and multiple regressions were conducted to identify independent predictors. A *p*-value of 0.05 was considered statistically significant.

## 3. Results

### 3.1. General Characteristics of the Study Population

After applying the inclusion and exclusion criteria, 202 patients were included in the final study. Most of the included patients were males (74.25%), living in a rural environment (58.42%). The mean age at diagnosis was 66.62 ± 9.14 years old.

Table 2 presents the detailed characteristics of the study population.

**Figure 1:**
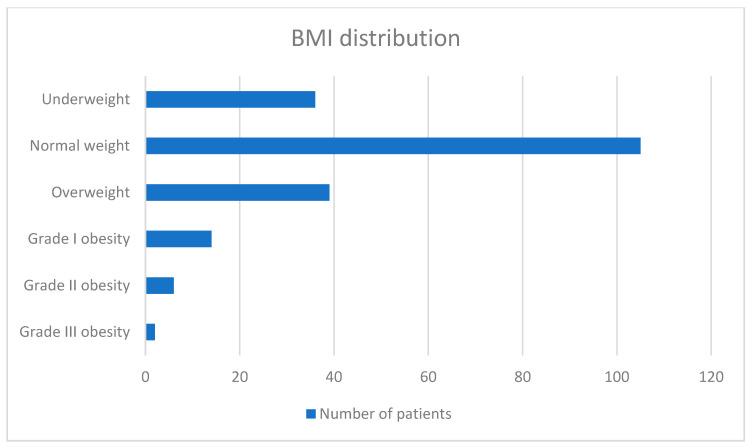
BMI distribution of cancer patients.

In addition, as Figure 1 depicts, most patients were of normal weight (51.98%). 86.63% of patients were active smokers, and 40.1% of them had an associated diagnosis of COPD, the most common comorbidity encountered in our study.

### 3.2. Histological Types of Lung Carcinoma and Stage at Diagnosis

**Figure 2:**
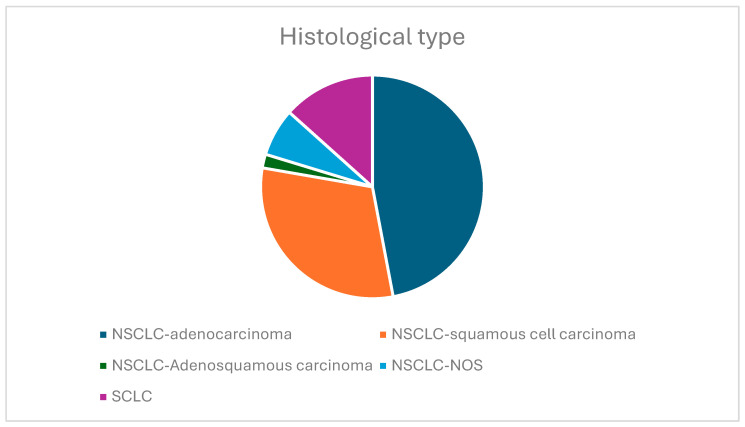
The histological type of lung carcinoma.

Figure 2 shows the histological classification of the included patients. Of the 202 patients included in the study, 27 were diagnosed with SCLC and 175 with NSCLC. In addition, from the total of 175 patients diagnosed with NSCLC, 95 were diagnosed with adenocarcinoma, 62 with squamous carcinoma, 14 with Not Otherwise Specified NSCLC (NOS-NSCLC), and 4 with adenosquamous lung carcinoma.

**Figure 3:**
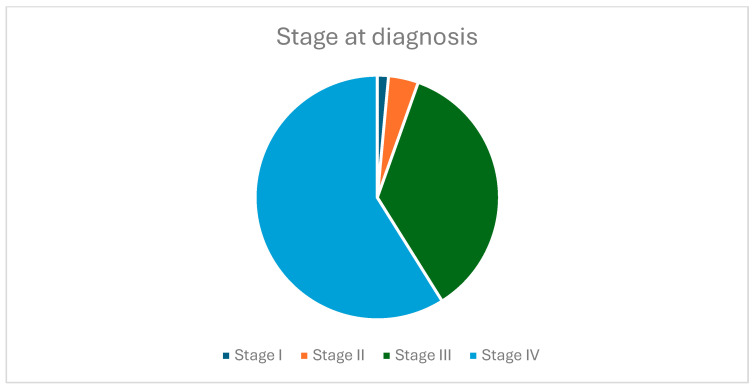
Tumor stage at diagnosis.

Figure 3 presents the tumor stage at the time of the initial diagnosis. Of the 202 patients included in the study, 3 were diagnosed in stage I, 8 in stage II, 72 in stage III, and 119 in stage IV. The stage at diagnosis was established based on the eighth edition of the TNM system.

**Figure 4:**
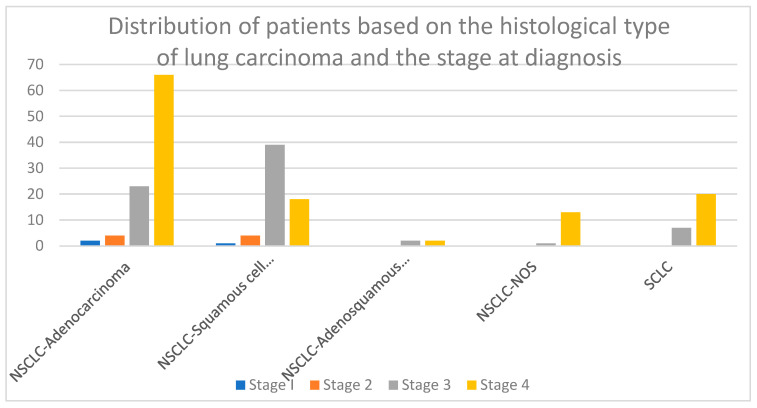
Distribution of patients based on histological type of lung carcinoma and stage at diagnosis.

In addition, Figure 4 provides detailed data regarding the relationship between the tumor stage at diagnosis and its histological type in all 202 patients included.

### 3.3. CBC-Derived Predictors of Severity in Lung Cancer

A multivariate regression was performed to identify the independent predicting factors of severity for all the histologic subtypes of lung cancer included in this study.

The first step was to identify the factors that predicted the severity of the two main histologic lung carcinoma types: NSCLC and SCLC.

For NSCLC patients, the leukocyte count (*p* = 0.01), the neutrophil count (*p* = 0.011), and male gender (*p* = 0.014) were found to be significant predictors of severity, while for SCLC patients many more parameters were identified. Male gender (*p* = 0.0002), COPD (*p* = 0.0001), abnormal levels of four cellular lines, and seven CBC-derived inflammatory indexes could predict severity in SCLC patients. Among the included inflammatory markers, AISI (*p* < 0.0001), SII (*p* < 0.0001), SIRI (*p* < 0.0001), d-NLR (*p* < 0.001), EMR (*p* = 0.004), ENR (*p* = 0.004), and MLR (*p* = 0.001) reached the statistical relevance as severity predictors in SCLC patients.

**Figure 5:**
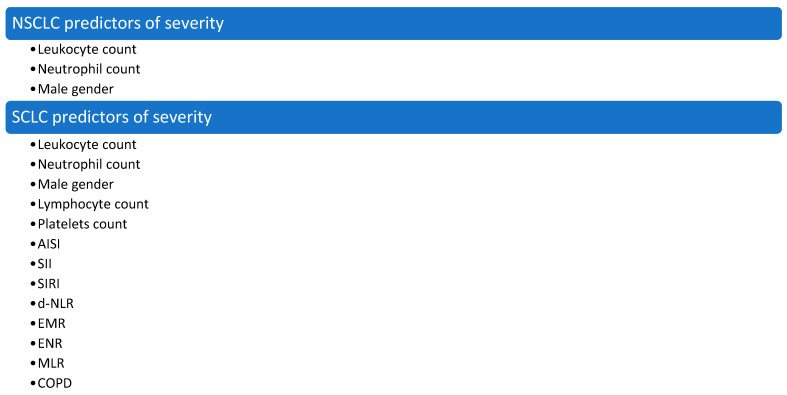
Predictors of severity for both NSCLC and SCLC patients.

The differences between the different predictors of severity between NSCLC and SCLC patients are summarized in Figure 5.

The second step of the study was to perform the multivariate regression for the different histologic subtypes of NSCLC, with the final goal of identifying both similarities and differences between the inflammatory response encountered in squamous cell carcinoma, adenocarcinoma, adenosquamous carcinoma, and NOS-NSCLC.

**Figure 6:**
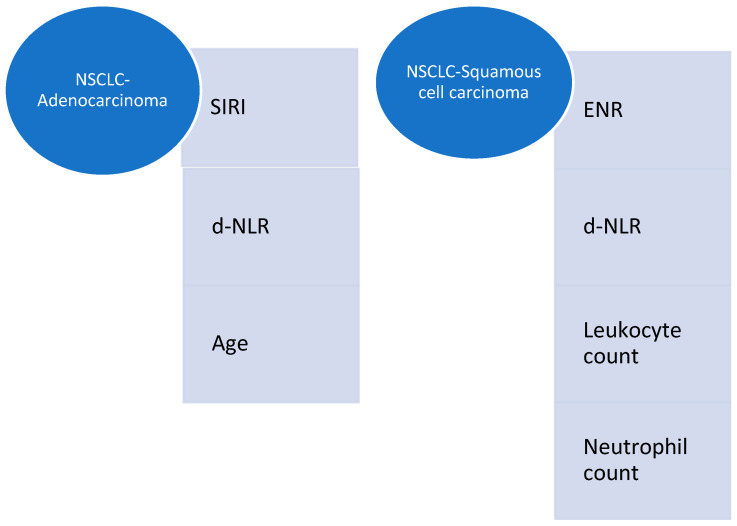
Differences between predictors of severity in adenocarcinoma and squamous cell carcinoma patients.

The results showed that inflammation varies according to the histologic subtype of NSCLC. The results can be consulted in Figure 6, which presents the differences between the predictors of severity in the two main histologic subtypes of NSCLC.

For adenocarcinoma patients, SIRI (*p* = 0.005), d-NLR (*p* = 0.039), and the patient’s age (*p*: 0.004) were significant predictors of severity.

For patients diagnosed with squamous cell carcinoma, d-NLR (*p* = 0.02) was also found to predict severity, in addition to ENR (*p* = 0.004), the leukocyte count (*p* = 0.03), and the neutrophil count (*p* = 0.01).

To better understand the detailed predictors of severity in NSCLC and SCLC patients, we summarized the results and their associated statistical significance in Table 3.

### 3.4. A Summary of Correlations Found Between the CBC-Derived Inflammatory Markers, the Stage of the Tumor, and the General Characteristics of the Patients

The final step of the study was to test for correlations between the CBC-derived inflammatory parameters of the included patients, the stage of the tumor at diagnosis, and the general characteristics of the study population (age, gender, BMI, living area, smoking, and COPD) for each histological subtype.

The results showed multiple statistically significant results for both NSCLC and SCLC patients, which are detailed in Table 4. 

As presented in Table 4, in patients diagnosed with adenocarcinoma, the PLR levels were positively correlated with the stage of the disease at diagnosis (*p* = 0.0266). At the same time, we found a negative association between the platelet count and BMI (*p* = 0.0268). In addition, for adenocarcinoma patients, active smoking was correlated with an increased risk of COPD (*p* = 0.0425) and an increased inflammatory status, as the d-NLR (*p* = 0.0127), NLR (*p* = 0.0443) and SII levels (*p* = 0.0328) were all higher in smokers compared to non-smokers.

In squamous cell carcinoma patients, we found a positive correlation between the BMI and COPD (*p* = 0.0301) and a negative one between the BMI and d-NLR (*p* = 0.0382) levels.

For patients diagnosed with NSCLC-NOS, male gender was correlated with smoking (*p* = 0.0075). At the same time, a negative correlation between the BMI and SII levels was depicted (*p* = 0.0425).

For SCLC patients, smoking was found to be associated with male gender (*p* = 0.0413).

## 4. Discussion

The discovery of effective, inexpensive, and easily achievable biomarkers for the early identification of high-risk lung cancer patients represents a key point in the management of this highly deadly condition.

High levels of CBC-derived inflammatory indexes were associated with an increased risk of developing chronic pathologies, including peritoneal dialysis-associated peritonitis [31]. They were also cited as potential predictors for NSCLC and other malignancies, as inflammation plays an essential part in tumor progression and is one of the hallmarks of cancer [27].

As presented in Table 4, the current study confirmed that CBC-derived inflammatory indexes, along with some key cellular line alterations, act as severity predictors in both SCLC and NSCLC patients. We identified similarities and differences between the inflammatory response of SCLC and NSCLC patients, the main one being related to the highly increased number of CBC-derived inflammatory indexes encountered in SCLC patients compared to NSCLC patients.

The results of our study are supported by previous findings, as NLR, d-NLR, PLR, and SII were identified to be associated with an increased risk of developing solid cancers [21,32,33,34,35]. High NLR and PLR levels were cited as potential predictors for the prognosis and overall survival in multiple malignancies, like melanoma, renal cell carcinoma, gastric cancer, and NSCLC [36,37,38,39]. A study published by Cupp et al. in 2020 linked high NLR levels with a worse prognosis for cancer patients [40], while Liu et al. stated that low NLR and PLR values in NSCLC patients were correlated with better outcomes [21]. In addition, elevated NLR levels were associated with a worse overall survival in both NSCLC and SCLC [23]. A study published by Wuhao Huang et al. in 2018 also mentioned that the combination of NLR levels and preoperative fibrinogen can act as an independent prognostic indicator for disease-free survival (DFS) and OS in resectable NSCLC [41]. Furthermore, the use of d-NLR for the improvement of the clinical outcome of lung cancer patients was also addressed by Kuang et al. in 2024, who stated that d-NLR has been shown to act as a predictive biomarker for assessing the effects of therapies for SCLC patients. Their results showed that lower d-NLR levels were associated with a better response to immunotherapy regimens [42]. In addition, NLR can also be estimated using DNA methylation data as methylation-derived NLR (mdNLR). This topic was explored by Zhao et al. in 2021 in their study, as they explored the association between mdNLR and lung cancer risk. Their results showed that in NSCLC patients, for one standard deviation increase in mdNLR levels, the risk of dying from lung cancer increased by 50%, while the risk of developing NSCLC also increased by 47% for the same increase in the standard deviation [43].

In regard to the MLR levels, they were cited as independent predictive biomarkers in patients with surgically treated lung cancer [44].

In addition, the histologic characteristics of tumors might impact the clinical management of cancer patients, as histology can guide treatment and predict the prognosis based on associated risk factors and genetic disorders.

The topic of histology in lung cancer prognosis is still understudied and remains an essential part of the carcinogenesis processes. Hirsch et al. performed extensive literature research to assess histology’s prognostic or predictive value in lung cancer patients. Findings showed that histology may influence the efficacy of different chemotherapeutic regimens, as adenocarcinoma patients treated with EGFR tyrosine kinase inhibitors were shown to have better disease control and treatment response rates than patients diagnosed with squamous cell carcinoma or other non-adenocarcinoma subtypes [45]. The topic was also addressed by Hoang et al., who assessed the relationship between the histologic subtypes of NSCLC and the survival rates of patients treated with platin-based chemotherapeutic drugs [46]. Their results showed no difference regarding the survival, performance status, or the degree of weight loss among the four histology groups assessed in the study (adenocarcinoma, squamous cell carcinoma, NOS, and large cell carcinoma). Additional studies showed that adenocarcinoma was associated with superior response rates, disease control rates, progression-free survival, and survival in patients with advanced NSCLC. In contrast, squamous cell carcinoma was associated with a shorter survival in NSCLC patients treated with cisplatin [45].

Although inflammation and histology are essential findings in the development of malignant processes, we still lack information regarding histology’s impact on the patient’s inflammatory status. Therefore, this study was designed to identify those specific CBC-derived inflammatory biomarkers that can be used as independent predictors of severity, considering the histology of lung carcinoma. The main goal was to acknowledge common and distinct characteristics of tumor-induced inflammation in the major histologic types of NSCLC and SCLC and relevant correlations between histology findings and the included patients’ general and inflammatory factors.

Based on our findings, the histologic type of lung cancer may influence the inflammatory response in lung cancer patients by impacting different cellular lines and inflammatory indexes. On one hand, when comparing the CBC-derived inflammatory parameters that can serve as predictors of severity in NSCLC and SCLC patients, we observed that the leukocyte count and neutrophil count were common predictors of severity in these two major histologic subtypes of lung cancer. On the other hand, in SCLC patients, the number of CBC-derived inflammatory indexes that could predict severity was much higher than in NSCLC patients, as AISI, SII, SIRI, d-NLR, EMR, ENR, and MLR were also identified as predictors of severity. In addition, the leukocytes, neutrophils, lymphocytes, and platelet count could predict the severity of lung cancer patients diagnosed with SCLC, similarly to an associated diagnosis of COPD. Previous studies support our results up to a certain point, as the relationship between cancer and different cellular lines is considered an emerging direction in carcinogenesis. Virchow was the first scientist to notice leukocytes within the tumors [47]. Besides leukocytes, multiple cellular lines were proposed in recent years as essential in cancer progression. The number of leukocytes, neutrophils, platelets, and lymphocytes varies during the process of tumoral growth and lysis, due to the associated secretion of cytokines [48,49].

Eosinophils have been cited as promising cells in cancer progression and prognosis. The influence of the eosinophil count in lung cancer patients needs to be further studied, as eosinophils might play a key role in the inflammation associated with malignant tumors. Distinct data regarding the role of eosinophils in the prognosis of cancer patients are found in the literature, as recent studies have linked eosinophils with both immunoinhibitory and immunostimulatory functions in tumor development [50]. Until now, studies have explored the link between eosinophils and NSCLC, but few have reported the association between eosinophils and SCLC. Studies conducted by Ownby et al. and Prizment et al. linked a high eosinophil count with a better prognosis for cancer patients [51,52]. Interestingly, the current study did not find a correlation between eosinophils and NSCLC but rather between eosinophils and their impact on SCLC severity. We observed that the EMR and ENR levels are predictors of severity in SCLC patients and are not associated with NSCLC severity. This suggests that eosinophil variations might be involved in the augmented inflammatory response encountered in SCLC patients. Further studies are needed to confirm the exact relationship between the eosinophil count, eosinophil-derived inflammatory indexes, and their involvement in cancer pathogenesis.

The current study revealed key points regarding the different inflammatory responses encountered in the two major histologic types of lung cancer. Inflammation might play an essential role in the poorer outcomes of patients with SCLC, as multiple cellular lines and inflammatory indexes were described as predictors of severity in this histologic type of lung carcinoma compared with NSCLC.

The number of patients diagnosed with NSCLC was much higher compared to the SCLC ones. NSCLC is diagnosed about nine times more frequently than SCLC [53,54]. In our study, the number of patients diagnosed with NSCLC was approximately 6.5 times higher than those diagnosed with SCLC, supporting that NSCLC is much more frequently diagnosed than SCLC. It is known that patients with a diagnosis of SCLC have higher mortality rates, a risk of metastasis, a decreased quality of life, and a decreased survival rate. Based on our findings, increased inflammation could explain the difference in the prognosis between SCLC and NSCLC patients, as increased inflammation is associated with worse survival and overall prognosis [54].

In addition, lung cancer outcomes could be influenced by gender, as studies showed that women might have an increased survival rate compared to men, mainly due to the higher tobacco exposure in men. A study published by Visbal et al. estimated a 5-year survival rate of 15% in men and 19% in women diagnosed with SCLC [55]. Our study confirms these findings, as male gender was identified to be a predictor of severity in both NSCLC and SCLC patients.

As the histologic type of lung cancer might impact the patient’s outcome, the current study aimed to assess relevant inflammation-based variations encountered between the histologic subtypes of NSCLC to better assess NSCLC patients. Results showed that d-NLR could predict severity in both adenocarcinoma and squamous cell carcinoma patients. In addition, for adenocarcinoma patients, SIRI and an older age at diagnosis acted as specific predictors of severity. At the same time, in squamous cell carcinoma, ENR, the leukocyte count, and the neutrophil count were identified as particular predictors of severity. Our findings showed that although adenocarcinoma and squamous cell carcinoma are both NSCLC tumors and share some standard histologic features, they are, in fact, driven by a different tumor-associated inflammatory response. Understanding these differences in terms of inflammatory variations might guide the clinical approach for patients and provide specific therapies, taking into account the particular characteristics of the tumor microenvironment. These might represent key findings for improving the quality of life and, ultimately, the overall prognosis of lung cancer patients.

The relationship between the BMI and lung cancer prognosis is still under debate. Previous studies reported that a higher BMI (>25 kg/m^2^) is associated with a better long-term survival and might act as an independent predictor of better treatment outcomes and a lower risk of developing lung cancer [44,56,57,58,59,60,61]. In contrast, other studies mentioned that obesity might increase the risk of being diagnosed with lung adenocarcinoma [62]. In addition, obesity might contribute to chronic inflammation and promote cancer progression [63]. Surprisingly, our study identified a negative correlation between the patients’ BMI and platelet number in adenocarcinoma patients (*p* = 0.0268), BMI and d-NLR levels in squamous cell carcinoma (*p* = 0.0382), and BMI and SII levels in NSCLC-NOS patients (*p* = 0.0425). These findings might suggest that a higher BMI could be linked with a decreased inflammatory response in NSCLC patients, with obesity acting up to a certain point as a protective factor regarding the inflammatory response associated with some histologic types of NSCLC. These findings are consistent with the latest term of the “obesity paradox”, as obesity might partially act as a protective factor that lowers the risk of recurrence and death in lung cancer patients [64]. Increased adipose tissue might lead to an increased secretion of anti-inflammatory substances, like adipokines with immunomodulatory properties [65]. In addition, obese patients can better tolerate cancer treatments and, therefore, up to a certain point, will have an improved prognosis [66]. More research is needed to fully understand the involvement of adipose tissue in regulating the inflammatory response in lung cancer patients, especially with an increased focus on histologic types, specific cellular lines, and CBC-derived inflammatory parameters.

It is known that smoking increases the risk of lung cancer, causing inflammation, cellular destruction, and decreased blood flow. Oxidative stress with high levels of reactive oxygen species (ROS) can lead to mucus hypersecretion, lung inflammation, the inactivation of anti-protease, DNA damage, and the induction of carcinogenesis [67]. In the current study, 86.63% of patients were active smokers, and 40.1% of the study population had COPD as the main comorbidity. Our study showed that smoking was associated with increased inflammatory changes only in adenocarcinoma patients, as the d-NLR (*p* = 0.0127), NLR (*p* = 0.0443), and SII (*p* = 0.0328) levels were higher in smoker adenocarcinoma patients. In squamous cell carcinoma and adenosquamous carcinoma, no correlation between smoking and any inflammatory parameters was found, while in NSCLC-NOS and SCLC patients, smoking was associated with male gender (*p* = 0.0075). These findings might suggest that although smoking induces local and systemic inflammatory changes, there are still unknown factors that link inflammation and cigarette smoking, with histology being one of them. Our findings differ from data found in the literature, as previous studies reported that the strongest association with smoking was found in squamous cell carcinoma [45], a hypothesis disproved by the current research.

Regarding COPD, it was found to be an independent predictive factor for severity in SCLC patients. Interestingly, COPD was not found to predict severity in NSCLC patients in general or in squamous cell carcinoma or adenocarcinoma patients precisely. On the other hand, COPD was positively associated with smoking in adenocarcinoma patients (*p* = 0.0425) and with a higher BMI in squamous cell carcinoma patients (*p* = 0.0301), raising awareness about the combination of COPD, obesity, and smoking and its predictive value for lung severity. The combination of COPD and smoking increases the mortality rates among lung cancer patients, as the co-existence of both COPD and cancer is ten times higher in smokers than in non-smokers. Analyzing relevant data from the literature, we found that the relationship between COPD and lung cancer is still under debate, with tobacco exposure, DNA damage, genetic abnormalities, and inflammatory changes cited as the main common pathophysiological factors involved in this process [66,67]. In addition, free radicals lead to the activation of intracellular signals and promote angiogenesis and mitosis, leading to an inflammatory-induced uncontrolled cellular and vascular proliferation of the tumor [3].

From our knowledge, this is the first study in which nine CBC-derived inflammatory indexes were analyzed and correlated with the histological subtype of lung carcinoma and other general data about the patient. The present study demonstrates key points regarding the different CBC-derived inflammatory predictors of severity and the histologic subtypes of lung cancer, raising awareness about the importance of histology and inflammation in properly assessing and managing cancer patients. Our research aims to be one of the first steps needed for a better understanding of the complexity of immune interactions between different cellular lines and inflammatory parameters derived from the CBCs of patients. The key to improving treatment regimes and the overall prognosis in lung cancer patients might be related to the development of personalized medicine, target immunotherapy, and the early identification of high-risk patients. In this manner, patients could receive better treatments, reducing side effects and overall therapy costs. Nowadays, increased efforts are being made towards the same goal: the earlier identification of high-risk patients. Therefore, a recent study published by Wang et al. in 2024 proposed an interesting topic for diagnosing lung cancer relapse. They discussed the concept of the folate receptor-positive (FR^+^) circulating tumor cell count (CTC) in the progression of lung cancer patients. Circulating tumor cells are cells released into the peripheral blood of the patient, either from the primary tumor itself or from metastatic points. In addition, tumor cells need high amounts of folic acid during their DNA synthesis in order to properly proliferate. Therefore, integrating these results into clinical practice could improve the clinical outcome of this highly deadly condition. In addition, Wang et al. showed that using the combination between the FR^+^-CTC combined with the dNLR and lymphocyte count (LC) could aid in diagnosing relapse cases in lung cancer patients [68].

Nevertheless, this study has some limitations, starting with its retrospective nature. We lack information regarding patient follow-up, since multiple patients chose to continue their treatment regimes in other hospitals and cities. Without knowledge of these aspects, it is challenging to outline definitive conclusions regarding the impact of inflammation and histology on the overall survival of lung cancer patients. However, although several studies have addressed the relationship between inflammation and overall prognosis, few, so far, have addressed the complex interaction between easily achievable CBC-derived inflammatory parameters, histologic characteristics, and their impact on lung cancer patients’ prognosis.

Further extensive studies are needed to understand histology’s impact on the lung cancer prognosis and therapeutic options, as assessing different inflammatory parameter variations can lead to the development of personalized treatment regimens that target specific abnormal cellular lines for each histologic type of SCLC and NSCLC. Characteristics like immune cells, tumor infiltrative properties, the tumor microenvironment, angiogenesis, and DNA damage play a crucial role in tumor progression and carcinogenesis in general. The current study can be considered a pioneering study, as it enhances the idea that for each specific histological type, different parameters influence the inflammatory response and predict severity; therefore, these factors should be considered when managing both NSCLC and SCLC patients.

We intend to strengthen the key findings proposed in this article with future prospective studies to validate the utility of these markers in predicting, alongside severity, the treatment response and long-term outcomes.

## 5. Conclusions

As lung cancer remains a global health concern, with an increased rate of mortality, this study aimed to be one of the first steps that integrated and compared the inflammation associated with different histologic subtypes of lung cancer, with the final goal of establishing the complex relationship between chronic inflammation, histologic findings, and predicted lung cancer severity. An early diagnosis and accessible predictive biomarkers, alongside an improved clinical, radiological, and pathophysiological understanding of the particularities of each histologic subtype of lung cancer, represent emerging directions for improving the outcomes of lung cancer patients. Integrating easily achievable CBC-derived parameters into routine clinical workflows for lung cancer diagnosis and monitoring could improve the clinical management of these high-risk patients, as well as their quality of life and overall survival. In addition, the study of cellular lines, CBC-derived inflammatory indexes, and their interactions with histology and the tumor microenvironment might identify high-risk patients who primarily benefit from a personalized treatment and specific immunotherapy.

## Figures and Tables

**Table 1:** The formula of the analyzed CBC-derived parameters.

Parameter	Formula
Neutrophil-to-lymphocyte ratio (NLR)	Neutrophil count/lymphocyte count [×10^3^/μL] [1]
Derived-neutrophil-to-lymphocyte ratio (d-NLR)	Neutrophil count/(WBC − neutrophil count) [×10^3^/μL] [27]
Monocyte-to-lymphocyte ratio (MLR)	Monocyte count/lymphocyte count [×10^3^/μL] [27]
Platelet-to-lymphocyte ratio (PLR)	Platelet count/lymphocyte count [×10^3^/μL] [1]
Eosinophil-to-neutrophil ratio (ENR)	Eosinophil count/neutrophil count [×10^3^/μL] [28]
Eosinophil-to-monocyte ratio (EMR)	Eosinophil count/monocyte count [×10^3^/μL] [29]
Systemic inflammatory index (SII)	(Neutrophil count × platelet count)/lymphocyte count [×10^3^/μL] [19]
Systemic inflammatory response index (SIRI)	(Neutrophil count × monocyte count)/lymphocyte count [×10^3^/μL] [27]
Aggregate index of systemic inflammation (AISI)	(Neutrophil count × monocyte count × platelet count)/lymphocyte count [×10^3^/μL] [30]

**Table 2:** General characteristics of the study population.

Parameters	N (Absolute Count)	N (Percentage %)
ALL N = 202 patients		
AGE—mean: 66.62 ± 8.34		
<50 years	4	1.98%
50–59 years	37	18.32%
60–69 years	83	41.09%
70–79 years	71	35.15%
≥80 years	7	3.46%
Gender		
MALE	150	74.25%
FEMALE	52	25.75%
Living environment		
RURAL	118	58.42%
URBAN	84	41.58%
BMI—a median of numeric values (when available): 24 [23.328–24.653]		
<18.5	36	17.82%
18.5–24.99	105	51.98%
25–29.99	39	19.30%
30–34.99	14	6.94%
35–39.99	6	2.97%
>40	2	0.99%
Associated COPD		
YES	81	40.1%
NO	121	59.9%
Smoking		
YES	175	86.63%
NO	27	13.37%

**Table 3:** Predictors of severity for SCLC and NSCLC patients.

Factor	*p*-Value	95% CI	logOR	Overall Predictive Value
	NSCLC patients			
Leukocyte count	0.01	0.004632–0.03408	0.019	*p* = 0.0034
Neutrophil count	0.011	0.003436–0.02620	0.014	
Male gender	0.014	0.02728–0.2383	0.13	
	NSCLC–Adenocarcinoma			
SIRI	0.005	0.06960–0.01266	−0.04	*p* = 0.0004
d-NLR	0.039	0.003848–0.1457	0.074	
Age	0.004	0.03392–0.006669	−0.02	
	Squamous cell carcinoma			
d-NLR	0.02	0.01410–0.1869	0.1	*p*= 0.0441
ENR	0.0004	−9.3075–−1.7811	−5.5	
Leukocyte count	0.03	0.01301–0.2771	0.15	
Neutrophil count	0.013	−0.3996–−0.05337	−0.23	
	SCLC			
AISI	<0.0001	−0.002430–0.001229	−0.00183	*p* = 0.0001
SII	<0.0001	0.001185–0.002034	0.00161	
SIRI	<0.0001	0.5917–1.1510	0.8713	
d-NLR	<0.001	−1.3360–−0.7728	−1.0544	
EMR	0.004	0.9183–3.8487	2.3835	
ENR	0.004	−48.5515–−11.9517	−30.25	
MLR	0.001	−8.1070–−2.7492	−5.4281	
COPD	0.0001	−1.0162–−0.4747	−0.7454	
Male gender	0.0002	−0.8465–−0.3574	−0.602	
Leukocyte count	<0.001	0.3202–0.5954	0.4578	
Lymphocyte count	0.0007	−1.2249–−0.4424	−0.8336	
Neutrophil count	<0.001	−0.6009–−0.3776	−0.4892	
Platelets count	0.042	−0.003261–−0.00007213	−0.00167	

**Table 4:** Significant correlations between the histological subtypes of lung cancer, CBC-derived inflammatory markers, and general characteristics of the patients.

NSCLC–Adenocarcinoma			
**Parameter**	**Correlation**	***p*-Value**	**Correlation Coefficient**
SII	Smoking	*p* = 0.0328	0.219
d-NLR	Smoking	*p* = 0.0127	0.255
PLR	Stage	*p* = 0.0266	0.227
Age	Gender	*p* = 0.0037	0.295
	Stage	*p* = 0.0096	−0.264
Gender	Age	*p* = 0.0037	0.295
Stage	PLR	*p* = 0.0266	0.227
	Age	*p* = 0.0096	−0.264
BMI	Platelets	*p* = 0.0268	−0.228
Smoking	COPD	*p* = 0.0425	0.209
	d-NLR	*p* = 0.0127	0.255
	NLR	*p* = 0.0443	0.207
	SII	*p* = 0.0328	0.219
COPD	Smoking	*p* = 0.0425	0.209
NSCLC-Squamous cell carcinoma			
BMI	COPD	*p* = 0.0301	0.273
	d-NLR	*p* = 0.0382	−0.262
Smoking	None	-	-
COPD	BMI	*p* = 0.0301	0.273
NSCLC–Adenosquamous carcinoma			
Gender	Living environment	*p* < 0.0001	1
Smoking	None	-	-
COPD	None	-	-
Living environment	Gender	*p* < 0.0001	1
NSCLC–NOS			
Gender	Smoking	*p* = 0.0075	0.679
BMI	SII	*p* = 0.0425	−0.548
Smoking	Gender	*p* = 0.0075	0.679
COPD	None	-	-
SCLC			
Gender	Smoking	*p* = 0.0413	0.395
Smoking	Gender	*p* = 0.0413	0.395
COPD	None	-	-

## Data Availability

No new data were created or analyzed in this study. Data sharing is not applicable to this article.

## Abbreviations

The following abbreviations are used in this manuscript:

CBC	complete blood count
NLST	National Lung Screening Trial
CT	computed tomography
SCLC	small-cell lung carcinoma
NSCLC	non-small cell lung carcinoma
SCCs	lung squamous cell carcinoma
LCCs	large cell carcinoma
AdCs	adenocarcinoma
CIS	carcinoma in situ
AAH	atypical adenomatous hyperplasia
AIS	adenocarcinoma in situ
KRAS	Kirsten Rat Sarcoma viral oncogene
EGFR	epidermal growth factor receptor
TIME	tumor immune microenvironment
IL-6	Interleukin 6
IL-1	Interleukin 1
DNA	deoxyribonucleic acid
NOS	not-otherwise-specified carcinoma
TNM	Tumor, Node, Metastasis
NLR	neutrophil-to-lymphocyte ratio
d-NLR	derived neutrophil-to-lymphocyte ratio
MLR	monocyte-to-lymphocyte ratio
PLR	platelet-to-lymphocyte ratio
ENR	eosinophil-to-neutrophil ratio
EMR	eosinophil-to-monocyte ratio
SII	systemic inflammatory index
SIRI	systemic inflammatory response index
AISI	aggregate index of systemic inflammation
COPD	chronic obstructive pulmonary disorder
BMI	body mass index
ROS	reactive oxygen species
MRI	magnetic resonance imaging
LDCT	low-dose CT scan
ADC	apparent diffusion coefficient
DWI	diffusion-weighted imaging
ACBS	Aarhus composite biomarker score
mGPS	Modified Glasgow Prognostic Score
CNG	Combined NLR and Glasgow Prognostic Score
PFS	progression-free survival
OS	overall survival
MdNLR	methylation-derived neutrophil-to-lymphocyte ratio
TBLB	transbronchial biopsy
PTLB	percutaneous transthoracic biopsy
FR^+^ CTC	folate receptor-positive circulating tumor cell count
LC	lymphocyte count
